# Airborne Benzo[a]Pyrene may contribute to divergent Pheno-Endotypes in children

**DOI:** 10.1186/s12940-021-00711-4

**Published:** 2021-04-09

**Authors:** Hyunok Choi, Miroslav Dostal, Anna Pastorkova, Pavel Rossner, Radim J. Sram

**Affiliations:** 1 College of Health, Lehigh University, Bethlehem, PA USA; 2 Department of Genetic Toxicology and Nanotoxicology, Institute of Experimental Medicine, Czech Academy of Sciences, Prague, Czech Republic

**Keywords:** Air pollution, Benzo[*a*]pyrene, Endotype;15-F_t2_-isoprostane, 8-oxo-7,8-dihydro-2′-deoxyguanosine

## Abstract

**Background:**

Asthma represents a syndrome for which our understanding of the molecular processes underlying discrete sub-diseases (i.e.*,* endotypes), beyond atopic asthma, is limited. The public health needs to characterize etiology-associated endotype risks is becoming urgent. In particular, the roles of polyaromatic hydrocarbon (PAH), globally distributed combustion by-products, toward the two known endotypes – T helper 2 cell high (Th2) or T helper 2 cell low (non-Th2) – warrants clarification.

**Objectives:**

To explain ambient B[*a*]P association with non-atopic asthma (i.e., a proxy of non-Th2 endotype) is markedly different from that with atopic asthma (i.e., a proxy for Th2-high endotype).

**Methods:**

In a case-control study, we compare the non-atopic as well as atopic asthmatic boys and girls against their respective controls in terms of the ambient Benzo[*a*]pyrene concentration nearest to their home, plasma 15-F_t2_-isoprostane (15-F_t2_-isoP), urinary 8-oxo-7,8-dihydro-2′-deoxyguanosine (8-oxodG), and lung function deficit. We repeated the analysis for i) dichotomous asthma outcome and ii) multinomial asthma—overweight/obese (OV/OB) combined outcomes.

**Results:**

The non-atopic asthma cases are associated with a significantly higher median B[*a*]P (11.16 ng/m^3^) compared to that in the non-atopic controls (3.83 ng/m^3^; *P*-value < 0.001). In asthma-OV/OB stratified analysis, the non-atopic girls with lean and OV/OB asthma are associated with a step-wisely elevated B[*a*]P (median,11.16 and 18.00 ng/m^3^, respectively), compared to the non-atopic lean control girls (median, 4.28 ng/m^3^, *P*-value < 0.001). In contrast, atopic asthmatic children (2.73 ng/m^3^) are not associated with a significantly elevated median B[*a*]P, compared to the atopic control children (2.60 ng/m^3^; *P*-value > 0.05). Based on the logistic regression model, on ln-unit increate in B[*a*]P is associated with 4.7-times greater odds (95% CI, 1.9–11.5, *P* = 0.001) of asthma among the non-atopic boys. The same unit increase in B[*a*]P is associated with 44.8-times greater odds (95% CI, 4.7–428.2, *P = 0.001*) among the non-atopic girls after adjusting for urinary Cotinine, lung function deficit, 15-F_t2_-isoP, and 8-oxodG.

**Conclusions:**

Ambient B[*a*]P is robustly associated with non-atopic asthma, while it has no clear associations with atopic asthma among lean children. Furthermore, lung function deficit, 15-F_t2_-isoP, and 8-oxodG are associated with profound alteration of B[*a*]P-asthma associations among the non-atopic children.

**Supplementary Information:**

The online version contains supplementary material available at 10.1186/s12940-021-00711-4.

## Introduction

Asthma represents the most common respiratory impairment worldwide, afflicting over 400 million people of all age groups, racial/ethnic backgrounds, and genders [1, 2]. The asthma burden is growing despite the introduction of therapeutic strategies, pharmacologic interventions, and other public health measures [2]. Asthma has come to be recognized as an umbrella term for multiple subtypes of diseases, originating from distinct mechanisms (i.e., endotype) [3–6]. However, in practice, asthma is still diagnosed based on observable clinical characteristics (i.e., phenotypes) [7]. Such ‘once-size-fits-all’ detection and management strategies, focused on symptom easement without considering the etiologic mechanisms, are thought to contribute to the burgeoning economic burden of asthma, particularly by those with poorly controlled subtypes [8]. Furthermore, nearly 50% of the poorly-controlled asthmatic children are expected to emerge as severe adult cases [9]. There is a growing call to diversify the asthma subtypes beyond allergic asthma, based on the cellular and molecular mechanistic origins [9]. Mechanism-based definitions of asthma could help identify early effect biomarkers for specific sub-types [7]. The development of such biomarker-based therapies in asthmatic children before the onset of irreversible respiratory injuries represents an urgent public health need [9, 10].

Asthma is presently distinguished in terms of two major endotypes, T helper 2 cell high (Th2-high) and T helper 2 cell low (non-Th2) [3, 5]. However, diagnostic criteria of Th-2 high and non-Th2 endotypes are both evolving and putative [10]. The Th2-high endotype is associated with clinical features such as early-onset atopy, IgE production, and eosinophilic inflammation [3]. Th2-high endotype is believed to be made up of at least three subtypes, early-onset atopic [11], late-onset eosinophilic, and aspirin-exacerbated respiratory disease (AERD) [10]. In contrast, the non-Th2 endotype is even less understood in terms of its mechanistic origin and progression, even though it is estimated to comprise about half of all asthma cases [12, 13]. The salient features of the non-Th2 endotype are that the sufferers are typically non-atopic, women, overweight/obese, or do not respond well to corticosteroid therapy [10]. It has also been associated with cigarette smoke exposure [10]. Another notable characteristic of the non-Th2 endotype is that oxidative stress likely mediates (i) initiation of the IL-17- pathways and (ii) dysregulation of innate immune cells, ultimately resulting in neutrophil activation [14].

To date, it remains unknown whether fossil-fuel combustion-based air pollution [15, 16], in particular, polyaromatic hydrocarbon (PAH), globally distributed combustion by-products contribute toward either Th2 or non-Th2 endotypes. In our earlier investigation, we have shown that childhood exposure to ambient Benzo[a]pyrene (B[*a*]P) is significantly associated with clinically diagnosed asthma in children [17–23]. In particular, ambient B[*a*]P exposure was associated with the largest increase in odds of asthma among the overweight/obese (OV/OB) children in a sexually dimorphic fashion [18]. However, we did not directly examine whether airborne PAH poses a greater risk on OV/OB girls via the non-Th2 endotype.

Here, we consider a collection of phenotypes (e.g., non-atopic and atopic asthma), clinical characteristics (e.g., sex, OV/OB status), biomarkers of molecular mechanisms (e.g., systemic oxidant burden), and natural history (e.g., lung function impairment) to contrast B[*a*]P association with non-atopic and atopic asthma cases, respectively. Specifically, we test a novel hypothesis that ambient B[*a*]P association with non-atopic asthma (i.e., a proxy of non-Th2 endotype) is markedly different from its association with atopic asthma (i.e., a proxy for Th2-high endotype). To distinguish the mechanistic insights underlying non-atopic and atopic asthma (i.e., phenotypes), we investigate the above hypothesis using three sub-hypotheses. First, we postulate that the B[*a*]P-asthma association is significantly different for the non-atopic children than the atopic children. Second, we posit that systemic oxidant burden (i.e.*,* plasma 15-F_t2_-isoprostane (abbreviated as 15-F_t2_-isoP) and urinary 8-oxo-7,8-dihydro-2′-deoxyguanosine (abbreviated as 8-oxodG)) and lung function deficit, as two respective biomarkers of endotype developments, play divergent roles in non-atopic vs. atopic asthma per unit increase in B[*a*]P exposure. Third, considering our earlier observations [18], we posit that asthma-BMI combined outcomes (i.e., OV/OB Control, Lean Asthmatic, and OV/OB Asthmatic) represent phenotypic spectra nested within the respective endotypes, compared to Lean Controls, the reference group. Thus, B[*a*]P association with the non-atopic OV/OB asthmatic girls are different from the B[*a*]P association with atopic OV/OB asthmatic girls.

## Material and methods

The study population’s socioeconomic traits, exposure sources, and overall approaches are described [18, 20, 23]. Briefly, a case-control study was conducted on 191 asthmatics and 194 control children in November 2008. Children were enrolled from an industrial city, Ostrava (*n* = 94 asthmatic and 96 controls), and semi-rural villages across the Southern Bohemia (*n* = 97 asthmatic and 98 controls) in the Czech Republic [24]. The children’s medical record, questionnaire data (filled out by the parents and the child’s primary care doctor), and informed consent are obtained. The children also provided blood and urine samples. The institutional review board at the Institute of Experimental Medicine, Academy of Science, Czech Republic, reviewed and approved the study.

### Study sites

A comparable number of case (*n* = 191) and control (*n* = 194) children were enrolled from case-control children were enrolled from an industrial city (i.e., Ostrava) and background towns (i.e., Southern Bohemia) in the Czech Republic [24]. Ostrava is a historically industrial city with a high level of coal mining activities, coal processing, and metallurgical refinement. The most polluted districts (i.e., Radvanice/Bartovice) within Ostrava was chosen as the target enrollment region. The district-level ambient mean (11.4 ng/m^3^) for B[*a*]P during our investigation period (November 2008) was approximately 11-times higher than the recommended ambient and indoor air quality standard (1 ng/m^3^) [25]. The mean outdoor B[*a*]P in our background region during the same period is 2.5 ng/m^3^. Known airborne B[a]P emission sources in the background region consist of residential heating and automobile exhaust [23].

### Air pollution monitoring of polycyclic aromatic hydrocarbons

Publicly available ambient concentration of Benzo[*a*]Pyrene (B[*a*]P) is downloaded from the Czech Hydrometeorological Institute for November 2008 [26]. Eight particle-bound PAHs are routinely monitored using Versatile Air Pollution Sampler [23, 27, 28]. CHMI manages and monitors eight airborne particle-bound PAHs and PM_2.5_ using Versatile Air Pollution Sampler (VAPS) since the 1990s [26]. Particle-bound PAHs are extracted from filters, and quantitative chemical analysis of PAHs was performed by high-performance liquid chromatography (HPLC) with fluorescence detection according to the US Environmental Protection Agency (EPA) method [26]. Based on the CHMI protocol, the mean daily level is measured once every three days for ten days/month in southern Bohemia; once every six days for five days/month in Ostrava. Additional details on quality assurance and control have been described [23, 27, 28].

### 15-F2t-IsoP immunoassay

Plasma concentrations of 15-F2t-IsoP are analyzed using immunoassay kits as described [29].

### Urinary 8-oxodG

The concentration of urinary 8-oxodG, using competitive ELISA, is performed [30]. Children’s urine sample are incubated with 50 μl of 8-oxo-dG standards (concentration range, 1.25–40 ng/ml), 50 μl of primary antibody (JaICA, Japan, clone N45.1, concentration 0.2 μg/ml) as well as 100 μl of secondary antibody conjugated with alkaline phosphatase. Any samples, which demonstrated inhibition < 20% or > 80% are repeatedly analyzed with or without additional dilution. Urinary 8-oxo-dG concentration is determined as nmol 8-oxo-dG/mmol creatinine.

### Asthma diagnosis

The diagnosis is made during multiple clinic visits, based on the child’s clinical symptoms, laboratory markers, and by performing lung function test, bronchodilation test, and skin prick tests on older children. The children’s designated primary care physician used a different set of clinical criteria for the older children (≥ 5 years in age) vs. infants and toddlers (≤ 4 years in age). In children 5 years in age or older, a positive diagnosis of asthma is made if the child had three or more of the following events, including the parental report of ≥2 episodes of cough (any time during the day); chest tightness and/or belabored breathing; wheezing and/or whistling sound during breathing; school absence or limitation in play activities; emergency room visit and/or hospitalization from acute symptom exacerbation. A skin prick test is given to each child to determine the allergen sensitization status (i.e.*,* wheal size ≥3 mm diameter) following an application of common local allergen on the volar arm (PhadiatopH, Pharmacia & Upjohn Diagnostics, Uppsala, Sweden). On children ≤ four years in age, the following primary and secondary criteria are considered. Major criteria include i) history of hospitalization for bronchiolitis or heavy wheezing dyspnea; ii) a minimum of three wheezing dyspnea episodes within the last six months; iii) positive asthma diagnosis in either parent, and iv) atopic dermatitis diagnosis. Minor criteria include i) atopic rhinitis diagnosis without an indication of infection; ii) history of wheezing without an indication of infection; iii) Eosinophilia; and iv) male sex. If the infant/toddler met at least one primary and at least two secondary criteria, he/she is considered to be at high risk of asthma. Such children are closely monitored.

On the other hand, asymptomatic controls included those who are free from all symptoms. For each new asthma case we identified, we recruited one control child who matches the case in terms of the geographic district, age range, and gender. All children were given a lung function test at the clinic following the American Thoracic Society and the European Respiratory Society guidelines [31]. The children who were positive for lung function deficit was defined as those with lower than expected FVC (< 85% of predicted based on age, sex, height, and weight for Czech children), lower than expected FEV1 (< 85% predicted), lower than expected PEF (< 75% predicted), or maximal mid-expiratory flow (< 75% predicted).

### Cotinine assays

Urinary concentration of creatinine-adjusted Cotinine concentration is measured using a spectrophotometric method [32], followed by radioimmunoassay [33]. A cut-off value of > 450 ng/mg and 20–449 ng/mg are used to define active and passive smokers, respectively [34].

### Statistical analysis

All of our analyses are stratified according to atopy status and gender to clarify the modification of B[*a*]P effects on asthma. We restricted the present analysis to those with low to no recent exposure to cigarette smoke (Cotinine < 20 ng/mg) by removing all active smoking (> 450 ng cotinine/mg creatinine) or passive smokers (20–449 ng/mg).

#### Exposure window of interest

Ambient B[*a*]P concentration at a monitor nearest to each child’s home was used as a proxy for his/her exposure. Consistent with our earlier analysis, we defined optimal exposure window as an average for 30-day periods during November 2008 [28, 35]. To better understand the representativeness of present exposure (during November 2008) for exposure during earlier childhood, we compared the median and interquartile ranges of the 30-day average for November 2008 against those from November 2007 and November 2006, respectively. The 30-day average concentration of November 2008 was consistent with the 30-day average concentrations from the earlier years.

#### Primary health outcomes of interest

As described earlier, each child was categorized into the lean, overweight, or obese category if their body mass index (BMI, weight/height^2^) fell within the expected range for European children at a given age and sex [36]. The overweight and obese were combined into a single category (and henceforth termed OV/OB) due to low count. We considered either asthma outcome alone (yes/no) or asthma diagnosis (yes/no) with OV/OB status (yes/no) combined outcome (with four categories). Set against the lean controls (i.e., the reference group), those with OV/OB outcome alone without asthma (i.e.*,* OV/OB control), lean asthmatics without OV/OB (i.e.*,* lean asthma), and the children with both OV/OB and asthma (i.e.*,* OV/OB asthma) are set as three nominal outcome groups.

#### Identification of confounders

In our earlier analysis, we examined potential confounding of B[*a*]P-asthma association by age, gender, Cotinine, the total number of smokers in the family, maternal smoking at home, body mass index, plasma levels of Vitamin C, A, and E (mg/L), the season of delivery (indicator variables, fall, winter, and spring), birth weight and gestational age at delivery. Either Pearson χ^2^ test or Spearman’s ranked agreement coefficient (δ) was used to consider the univariate association between each of the above variables with either ambient B[*a*]P or the asthma outcome, respectively [23]. The median and the interquartile range of the primary exposure and other covariates are compared across the outcome groups, using Jonkheere-Terpestra (JT) test with α = 0.05.

#### Model building

Parsimonious multinomial logistic regression models per one (ln)-unit increase in B[*a*]P are built by forward-selecting putative confounders, including Cotinine alone; cotinine and lung function deficit; Cotinine, lung function deficit, and 15-F_2t_-IsoP; or by adjusting for Cotinine, lung function deficit, 15-F_2t_-IsoP as well as 8-oxo-7,8-dihydro-2′-deoxyguanosine (8-oxodG) (Table 3 and S1-S4). Due to the high correlation with the plasma concentration of 15-F2t-IsoP, Carbonyl’s plasma concentration is not included in the final model as a covariate. Our regression diagnostics carefully examined the robustness of the estimated odds ratios following the removal of an influential variable. All analyses were performed with SPSS version 20.0.1 for Windows (SPSS Inc., Chicago, Illinois, USA).

## Results

### A descriptive comparison of non-atopic vs. atopic asthma cases and controls

Table 1 summarizes the demographic, exposures, and natural histories of non-atopic and atopic asthma cases, compared to their respective controls. The non-atopic asthma cases are associated with a significantly higher median B[*a*]P (11.16 ng/m^3^) compared to that in the non-atopic controls (3.83 ng/m^3^; JT *P*-value < 0.001). In contrast, atopic asthmatic children (2.73 ng/m^3^) were not associated with a significantly elevated median B[*a*]P, compared to the atopic control children (2.60 ng/m^3^; JT P-value > 0.05). Prevalence of OV/OB status was 3-times higher among the non-atopic asthmatic children (24%) compared to that in the non-atopic controls (8%; *P*-value < 0.05). In contrast, the prevalence of OV/OB asthma was about 2-times higher among the atopic asthmatic children (26%), compared to that among the atopic controls (14%; P-value > 0.05). The lung function deficit was comparably high in both non-atopic asthmatic (19%; *P*-values < 0.05) and atopic asthmatic (24%; P-values > 0.05) children, compared to their respective controls (0% in both control groups).

**Table 1:** Demographic and exposure traits in the sample, restricted to non-smoking children (i.e., urinary Cotinine ≤20 ng/mg)

	Non-atopic Control		Non-atopic Asthmatics				Atopic Control		Atopic Asthmatics	
	*N = 121* Median (Interquartile Range)		*N = 37* Median (Interquartile Range)				*N = 14* Median (Interquartile Range)		*N = 89* Median (Interquartile Range)	
Benzo[a]pyrene (ng/m^3^)	3.83 (2.27, 5.45)		**11.16 (8.64, 16.00)**			**	2.60 (1.80, 3.20)		2.73 (1.80, 8.64)	
corticoid treatment (months)	0 (0, 0)		**20 (0, 45)**			**	0 (0, 2)		**36 (8, 61)**	†
Cotinine / creatinine (μg/g)	7.32 (4.95, 11.63)		7.70 (4.68, 9.91)				7.68 (3.49, 11.28)		6.27 (4.46, 9.37)	
15-F2t-isoP (pg/ml plasma)	150.14 (128.02, 170.64)		151.23 (113.31, 170.82)				140.03 (119.69, 164.35)		148.79 (135.29, 169.90)	
8-oxodG (nmol/mmol creatinine)	5.87 (4.71, 7.40)		5.25 (4.41, 7.26)				4.17 (3.34, 6.04)		5.38 (4.39, 6.91)	
Carbonyls (nmol/ml plasma)	21.01 (18.47, 24.06)		20.36 (18.06, 22.60)				19.82 (17.44, 23.54)		20.15 (18.02, 23.46)	
Age at first diagnosis (months)	127**‡** (26, 165)		17 (4, 50)				61**‡** (13, 107)		31 (6, 67)	
	***N***	***(%)***	***N***	***(%)***		***N***	***(%)***	***N***	***(%)***	
Sex (female)	58	(48%)	17	(46%)		6	(43%)	34	(38%)	
BMI category (Overweight/Obese)	10	(8%)	**9**	(**24%)**	†	2	(14%)	23	(26%)	
Lung function deficit (positive)	0	(0%)	**7**	(**19%)**	**	0	(0%)	**21**	**(24%)**	
Allergic rhinitis (yes)	2	(2%)	3	(8%)		7	(50%)	45	(51%)	
Atopic Dermatitis (Yes)	3	(3%)	**20**	(**54%)**	**	5	(36%)	32	(36%)	
Upper Respiratory Infection (Yes)	0	(0%)	2	(5%)		3	(21%)	13	(15%)	
Age group (adolescent)	61	(50%)	16	(43%)		5	(36%)	47	(53%)	
Maternal smoking at home (yes)	14	(12%)	4	(11%)		4	(29%)	13	(15%)	

The symbols ** and † denote *P*-values < 0.001 and 0.05, respectively. **‡** Sample sizes for non-atopic control and atopic control children are 4 and 13, respectively. Non-atopic and atopic asthma case sample sizes are 37 and 89, respectively. All categorical variables are tested using the Chi-square test of linear-by-linear association, and the continuous variables are tested using the Jonkheere-Terpestra test. All categorical variables are dichotomous and add up to 100%. The reference category, which are not shown, are as follows: sex (male), BMI (lean), lung function deficit (negative), allergic rhinitis (no), atopic dermatitis (no), upper respiratory infection (no), age group (pre-adolescent), and maternal smoking (no)

As shown in Table 1, the non-atopic and atopic children demonstrate an overall disparate clinical diagnosis pattern at ≤ three years of age. The non-atopic asthmatic children had their first clinical diagnosis around 17th months (IQR, 4–50 months; *P*-value > 0.05), compared to the non-atopic control, who were first diagnosed with any clinical outcome at 127th months (IQR, 26—165 months). Alternatively, the history of allergic rhinitis or upper respiratory infection at ≤ three years is most prevalent among the atopic control children (all *P*-values > 0.05; Table 1). In contrast, non-atopic asthmatics (54%) were associated with the highest prevalence of atopic dermatitis before age 3, compared to that in non-atopic controls (3%; P-value < 0.05).

### Sub-hypothesis 1: B[a]P-asthma association is significantly different for the non-atopic children and the atopic children

As shown in Table 2 (A), the highest ambient B[*a*]P exposure category (≥ 8.6 ng/m^3^) was associated with the highest prevalence of the non-atopic lean asthma (65%), compared to the prevalence in non-atopic controls (6%, *P-value < 0.001*). Among the non-atopic girls (Table 2 (A)), the highest ambient B[*a*]P exposure category (≥ 8.6 ng/m^3^) was associated with the highest increase in the prevalence of asthma (94%), compared to the non-atopic controls (10%; *P-values < 0.001*). In contrast, neither the atopic asthmatic boys nor the atopic asthmatic girls were associated with the highest B[*a*]P quartile exposure category, compared to their respective controls (both *P* > 0.05; Table 2 (A)).

**Table 2:** Children’s exposure to B[*a*]P exposure quartile categories and prevalence of asthma outcome (coded as dichotomous outcome (A) and nominal outcome (B)), stratified by the atopy- and gender-status of the children. Categorical variables are tested using Chi-square test of linear-by-linear association

			Non-atopic Children									Atopic Children								
(A)			***Controls***			***Asthmatics***						***controls***			***Asthmatics***					
			*N*	*(%)*		*N*			*(%)*		P-value	*N*	*(%)*		*N*		*(%)*		*P-value*	
	B[*a*]P (ng/m^3^) Exposure Quartile Categories																			
Boys	< 2.3		22	(35%)		2			(10%)		***<0.001***	1	(13%)		17		(31%)		*0.068*	
	2.3 - 4.2		11	(18%)		5			(25%)			7	(88%)		21		(38%)			
	4.3 - 8.5		26	(41%)		0			(0%)			0	(0%)		6		(11%)			
	≥ 8.6		4	(6%)		**13**			**(65%**)			0	(0%)		11		(20%)			
	Total		63	(100%)		20			(100%)			8	(100%)		55		(100%)			
Girls	< 2.3		11	(19%)		1			(6%)		***<0.001***	3	(50%)		14		(41%)		*0.066*	
	2.3 - 4.2		18	(31%)		0			(0%)			3	(50%)		4		(12%)			
	4.3 - 8.5		23	(40%)		0			(0%)			0	(0%)		2		(6%)			
	≥ 8.6		6	(10%)		**16**			**(94%**)			0	(0%)		14		(41%)			
	Total		58	(100%)		17			(100%)			6	(100%)		34		(100%)			
(B)		**Lean Control**		**OV/OB Control**			**Lean Asthma**		**OV/OB asthma**			**Lean Control**		**OV/OB Control**		**Lean Asthma**		**OV/OB asthma**		
		N	(%)	N		(%)	N	(%)	N	(%)		N	(%)	N	(%)	N	(%)	N	(%)	
	B[a]P (ng/m^3^) Exposure Quartile Categories																			
Boys	< 2.3	19	(34%)	3		(43%)	0	(0%)	2	(67%)	***0.020***	1	(14%)	0	(0%)	15	(35%)	2	(17%)	0.258
	2.3 - 4.2	8	(14%)	3		(43%)	4	(24%)	1	(33%)		6	(86%)	1	(100%)	15	(35%)	6	(50%)	
	4.3 - 8.5	25	(45%)	1		(14%)	0	(0%)	0	(0%)		0	(0%)	0	(0%)	5	(12%)	1	(8%)	
	≥ 8.6	4	(7%)	0		(0%)	**13**	**(77%**)	0	(0%)		0	(0%)	0	(0%)	8	(19%)	3	(25%)	
	Total	56	(100%)	7		(100%)	17	(100%)	3	(100%)		7	(100%)	1	(100%)	43	(100%)	12	(100%)	
Girls	< 2.3	10	(18%)	1		(33%)	1	(9%)	0	(0%)	***<0.001***	3	(60%)	0	(0%)	12	(52%)	2	(18%)	***0.013***
	2.3 - 4.2	17	(31%)		1	(33%)	0	(0%)	0	(0%)		2	(40%)	1	(100%)	3	(13%)	1	(9%)	
	4.3 - 8.5	22	(40%)		1	(33%)	0	(0%)	0	(0%)		0	(0%)	0	(0%)	1	(4%)	1	(9%)	
	≥ 8.6	6	(11%)		0	(0%)	10	(91%)	**6**	(**100%**)		0	(0%)	0	(0%)	7	(30%)	**7**	(**64%**)	
	Total	55	(100%		3	(100%)	11	(100%)	6	(100%)		5	(100%)	1	(100%)	23	(100%)	11	(100%)	

**Fig. 1:**
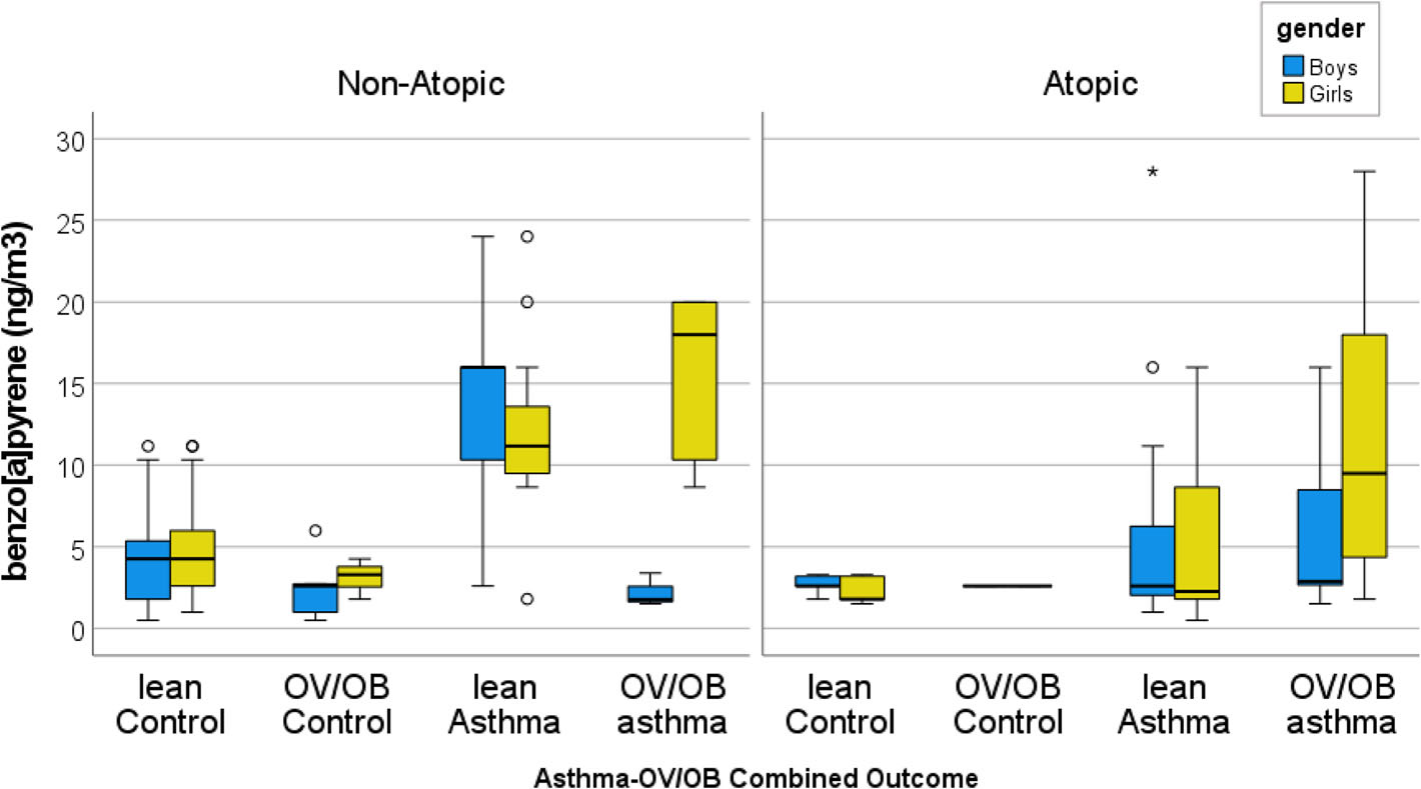
The box (i.e.*,* interquartile range and median) and whiskers (i.e., 5th and 95th percentile values) of ambient B[*a*]P concentrations for the boys (i.e., yellow bar) and the girls (i.e., green bar), according to the outcome categories (i.e., lean control, OV/OB control, lean asthma, and OV/OB asthma), and grouped according to gender and allergy status (non-atopic vs. atopic). The symbols ο and * represent values > 1.5– and > 3–fold, respectively, of the 75th percentile value

In particular, the highest B[*a*]P quartile exposure category was associated with the highest prevalence of the lean asthma outcome (77%) in non-atopic boys, compared to the non-atopic controls (7%, *P-value = 0.020*), and the OV/OB asthma outcome (100%) in the non-atopic girls, compared to the non-atopic control girls (11%, *P-value < 0.001*, Table 2 (B)). The same exposure category (≥ 8.6 ng/m^3^) was also associated with the OV/OB asthma outcome (64%) in atopic girls, compared to the atopic control girls (0%, *P-value = 0.013*, Table 2 (B)). In particular, non-atopic girls are associated with a step-wise increase in their median exposure to B[*a*]P (see Fig. 1). For example, the non-atopic girls with lean asthma were associated with 11.16 ng/m^3^, whereas the non-atopic girls with OV/OB asthmatic were associated with 18.00 ng/m^3^, compared to the non-atopic lean control girls (median, 4.28 ng/m^3^).

We account for the role of oxidative stress burden and lung function deficit in our final model (see Table 3 (A) and (B)). Based on the logistic regression model, on ln-unit increate in B[*a*]P is associated with 4.7-times greater odds (95% CI, 1.9–11.5, *P* = 0.001) of asthma among the non-atopic boys. The same unit increase in B[*a*]P is associated with 44.8-times greater odds (95% CI, 4.7–428.2, P = 0.001) of asthma among the non-atopic girls. In contrast, neither the atopic boys nor the atopic girls are associated with a significant increase in the odds of asthma per unit increase in B[*a*]P.

**Table 3.** Adjusted odds of asthma (coded as either dichotomous outcome (A) or multinomial outcome (B)) per unit increase in exposure to natural-log transformed B[*a*]P and stratified according to atopy- and sex-

	Main exposure	Main Health Outcome groups of interest								
		***Non-atopic Children***					***Atopic Children***			
**(A).**		***Control (Reference)***			***Asthmatics***		***Control (Reference)***			***Asthmatics***
		*N*			*N*		*N*			*N*
		OR (95% CI)			OR (95% CI)		OR (95% CI)			OR (95% CI)
		P-value			P-value		P-value			P-value
Male	one ln-unit B[a]P	*N = 63*			*N = 20*		*N = 8*			*N = 55*
		1			**4.7 (1.9―11.5)**		1			2.0 (0.4―11.1)
					***P =*** **0.001**					*P = 0.331*
Female	one ln-unit B[a]P	*N = 58*			*N = 1*		*N = 6*			*N = 34*
		1			**44.8 (4.7―428.2)**		1			3.0 (0.7―12.7)
					***P =*** **0.001**					*P* = 0.173
**(B).**		***Lean Control (Reference)***	***OV/OB Control***	***Lean Asthmatics***	***OV/OB Asthmatics***		***Lean Control (reference)***	***OV/OB Control***	***Lean Asthmatics***	***OV/OB Asthmatics***
		*N*	*N*	*N*	*N*		*N*	*N*	*N*	*N*
		OR (95% CI)	OR (95% CI)c	OR (95% CI)	OR (95% CI)		OR (95% CI)	OR (95% CI)	OR (95% CI)	OR (95% CI)
		*P-value*	*P-value*	*P-value*	*P-value*		*P-value*	*P-value*	*P-value*	*P-value*
Male	one ln-unit B[a]P	*N = 56*	*N = 7*	*N = 17*	*N = 3*		*N = 7*	*N = 1*	*N = 43*	*N = 12*
		1	0.3 (0.1**―**1.0)	**9.6 (2.9―32.1)**	0.3 (0.1**―**2.1)		1	0.4 (0.0**―**173.1)	1.6 (0.3**―**9.7)	2.2 (0.3**―**14.9)
			*P = 0.054*	***P < 0.001***	*P = 0.247*			*P = 0.786*	*P = 0.594*	*P = 0.409*
Female	one ln-unit B[a]P	*N = 55*	*N = 3*	*N = 11*	*N = 6*		*N = 5*	*N = 1*	*N = 23*	*N = 11*
		1	0.3 (0.0**―**2.9)	**27.4 (3.2―237.1)**	**46.1 (1.7―1271.4)**		1	0.0 (0.0**―**0.0)	3.6 (0.6**―**23.3)	**17.1 (1.8―165.6)**
			*P = 0.286*	***P = 0.003***	***P = 0.024***			*P = 0.998*	*P = 0.173*	***P = 0.014***

The dichotomous outcome model adjusts for Creatinine adjusted Cotinine, (ln) 15-F-2t-IsoP, (ln) 8-oxodG, and lung function deficit. The models adjust for Cotinine, lung function deficit, 15-F2t-IsoP, and 8-oxodG

### Sub-hypothesis 2: systemic oxidant burden and lung function deficit play divergent roles in non-atopic vs. atopic asthma per unit increase in B[a]P exposure

**Fig. 2:**
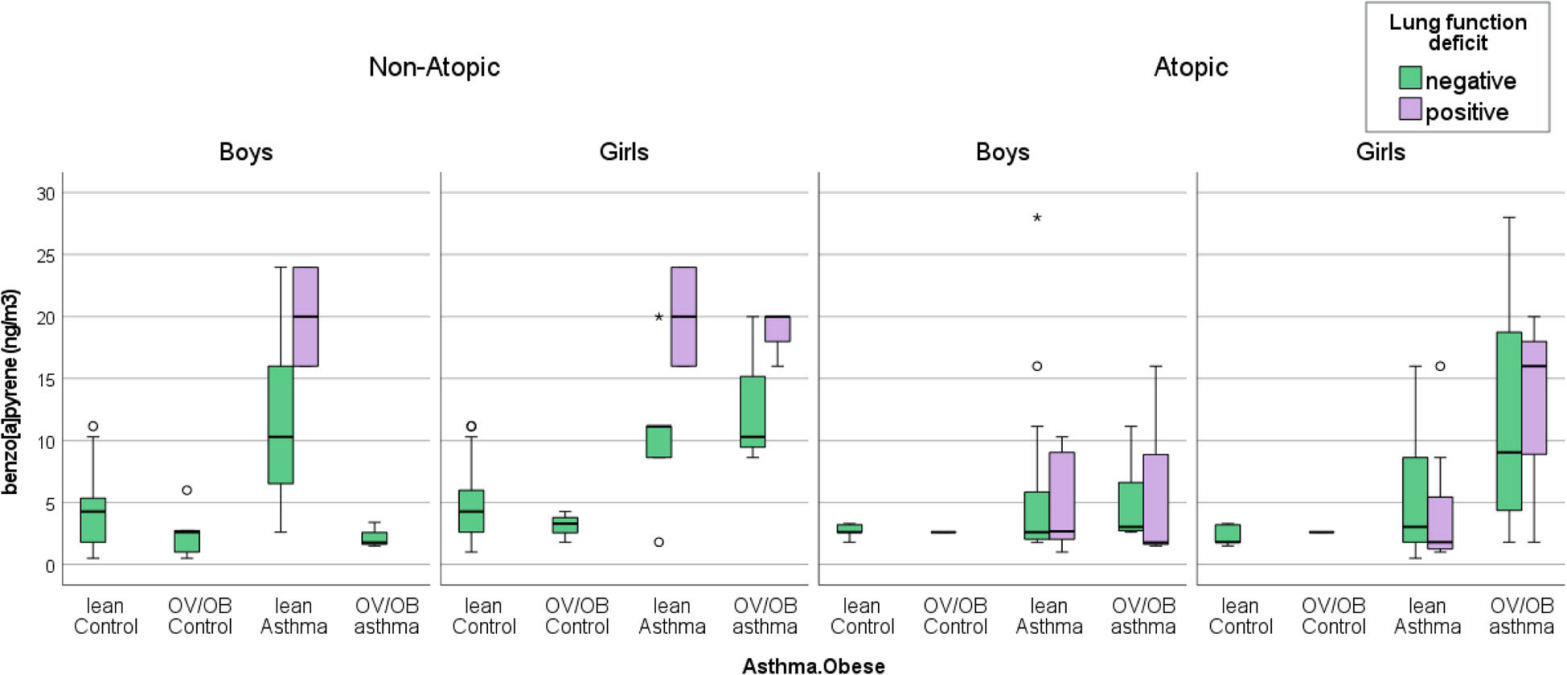
The box (i.e.*,* interquartile range and median) and whiskers (i.e., 5th and 95th percentile values) of ambient B[a]P concentrations for the health outcome groups (i.e., lean control, OV/OB control, lean asthma, and OV/OB asthma), in which each outcome is further stratified by a history of lung function deficit. The outcomes are presented according to gender (boys vs. girls) and atopy status (atopic vs. non-atopic). The symbols O and * represent concentrations > 1.5– and > 3–fold of the 75th percentile value

As shown in Fig. 2, the non-atopic boys with a lung function deficit diagnosis and lean asthma are associated with the highest median B[*a*]P (20.0 ng/m^3^; IQR, 16.0–24.5 ng/m^3^). A second-highest median B[*a*]P is observed in the non-atopic boys with lean asthma, but without the similar deficit (10.3 ng/m^3^; IQR, 2.7–16.0 ng/m^3^), compared to that for the controls (4.3 ng/m^3^; IQR, 1.8–5.4 ng/m^3^, Fig. 2). Similarly, the non-atopic girls with lung function deficit are associated the highest median B[*a*]P whether they had lean asthma (20 ng/m^3^; IQR, 16.0–24.0 ng/m^3^) or OV/OB asthma (20 ng/m^3^; IQR, 16.0–20.0 ng/m^3^), compared to that in the controls (4.3 ng/m^3^; IQR, 2.6–6.0 ng/m^3^; Fig. 2). In contrast, the non-atopic girls without the lung function deficit demonstrate a more modest increase in the median B[*a*]P among the lean asthmatics (11.2 ng/m^3^; IQR, 8.6–11.2 ng/m^3^) and OV/OB asthmatics (10.3 ng/m^3^; IQR, 8.6–15.0 ng/m^3^), compared to the non-atopic controls (Fig. 2).

Among the atopic boys, co-morbid presence of lung function deficit plus lean asthma (2.7 ng/m^3^; IQR, 1.9–9.3 ng/m^3^), or the lung function deficit plus OV/OB asthma (1.8 ng/m^3^; IQR, 1.5–9.3 ng/m^3^) is not associated with a different median B[*a*]P, compared to that in the atopic controls (2.6 ng/m^3^; IQR, 2.6–3.2 ng/m^3^; Fig. 2). Among the atopic girls, only those with OV/OB asthma as well as the lung function deficit are associated with a significantly elevated median B[*a*]P (16.0 ng/m^3^; IQR, 9.3–18.1 ng/m^3^), followed by those with OV/OB asthma without the deficit (9.1 ng/m^3^; IQR, 4.5–18.2 ng/m^3^), compared to the median in the atopic control girls (1.8 ng/m^3^; IQR, 1.7–3.3 ng/m^3^).

**Fig. 3:**
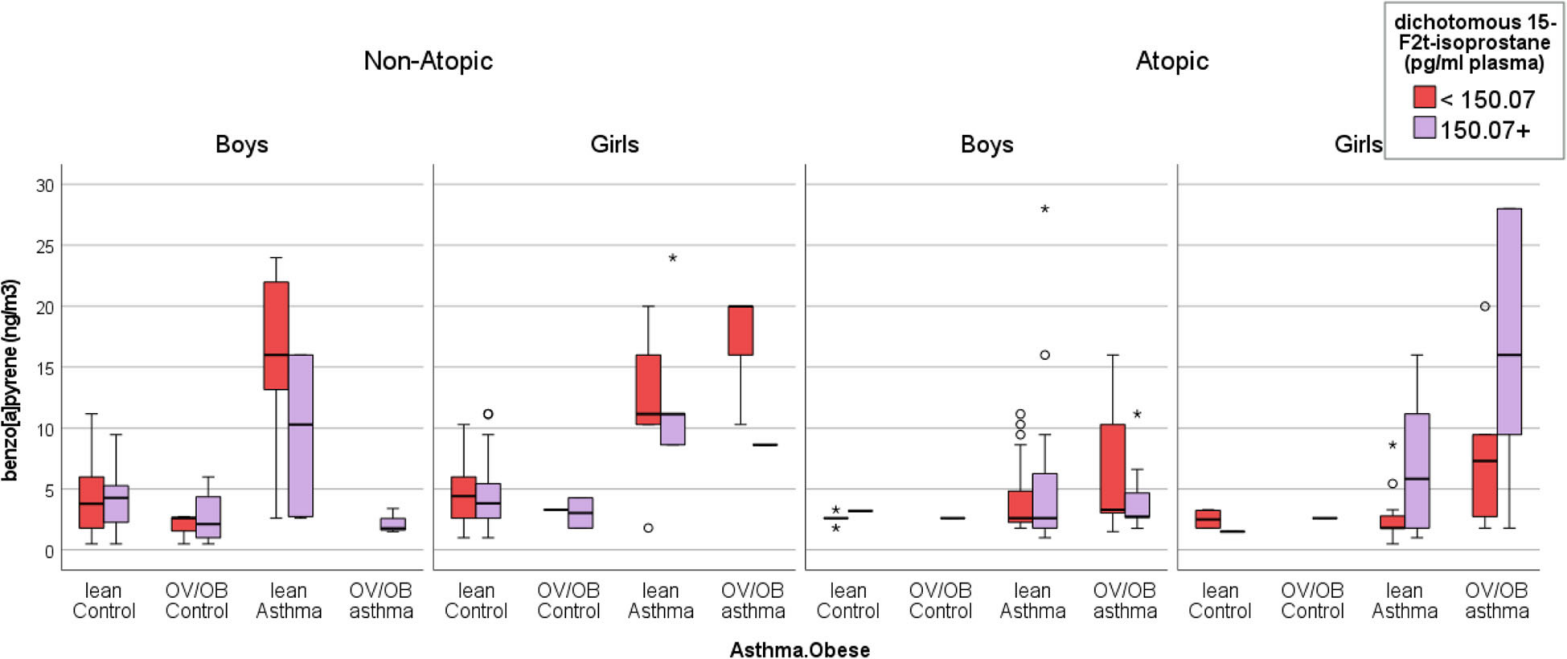
The box (i.e.*,* interquartile range and median) and whiskers (i.e., 5th and 95th percentile values) of ambient B[a]P concentrations for the health outcome groups (i.e., lean control, OV/OB control, lean asthma, and OV/OB asthma), in which each of the four outcomes is further stratified according to a median 15-F_2t_-IsoP value (150.07 pg/ml). Each panel represents a combined group, according to the atopy status (atopic vs. non-atopic) and gender (boys vs. girls). The symbols O and * represent concentrations > 1.5– and > 3–fold of the 75th percentile value

As shown in Fig. 3, the non-atopic versus the atopic asthmatic children are associated with an overall opposite trend in the relation between B[*a*]P and systemic oxidant stress marker levels (i.e., 15-F_2t_-IsoP and 8-oxodG levels, respectively). Among the non-atopic asthmatic boys and girls, those with the highest median B[*a*]P are associated with lower than the median 15-F_2t_-IsoP (< 150.07 pg/ml), compared to that in the respective non-atopic controls. For example, the non-atopic boys, a highest median B[*a*]P (16.0 ng/m^3^; IQR, 10.3–24.0 ng/m^3^) and the lean asthma outcome are observed among those with a lower than the median 15-F_2t_-IsoP, compared to B[*a*]P in the non-atopic control boys (median, 3.8 ng/m^3^; IQR, 1.7–6.0 ng/m^3^). Similarly, not only the non-atopic girls with the highest B[*a*]*P* value (median, 20.0 ng/m^3^; IQR, 13.0–20.0 ng/m^3^) and OV/OB asthma outcome but also those with a second-highest median B[*a*]P (11.2 ng/m^3^; IQR, 8.2–17.0 ng/m^3^) and lean asthma are both associated with lower than the median 15-F_2t_-IsoP (< 150.07 pg/ml), compared to the median B[*a*]P in the non-atopic control girls (median, 4.4 ng/m^3^; IQR, 2.6–6.0 ng/m^3^).

**Fig. 4:**
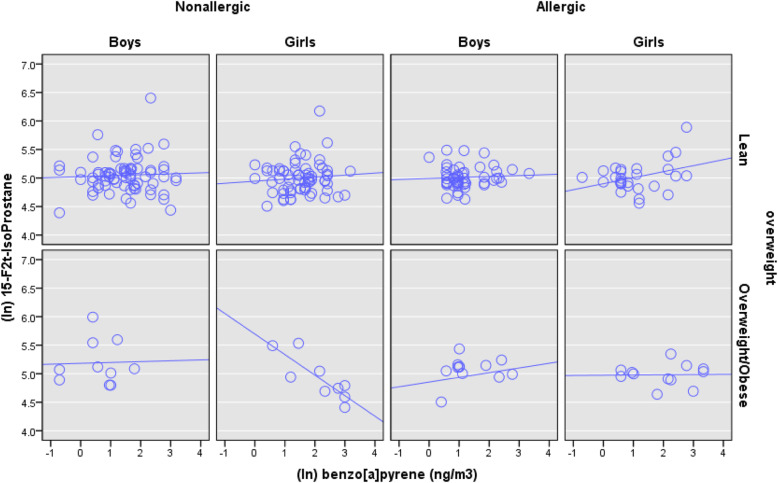
Scatter plot of natural log (ln)-transformed ambient B[a]P (ng/m^3^) and children’s natural log (ln)-transformed plasma 15-F_2t_-IsoP concentration. Each panel represents subgroups of children, who are stratified according to their atopy-, gender-, and OV/OB status. The open, blue circle represents the correlation point value for each child. The lines represent children’s plasma (ln) 15-F2t-IsoP as a linear function of (ln) B[a]P

In contrast, an opposite trend is observed among the atopic children. Namely, the atopic girls with lean asthma have not only a high median B[a]P (5.8 ng/m3; IQR, 1.8–11.2 ng/m3) but also a higher than the median 15-F_2t_-IsoP (Fig. 3). Furthermore, the atopic girls with OV/OB asthma not only have an even higher exposure to B[*a*]P (median, 16.0 ng/m^3^ IQR, 5.6–28.0 ng/m^3^) but also possess a higher than the median 15-F_2t_-IsoP value (≥ 150.07 pg/ml), compared to the B[*a*]P in the atopic control girls (median, 1.5 ng/m^3^ IQR, 1.5–1.5 ng/m^3^). Furthermore, the scatterplots (Fig. 4) of 15-F_2t_-IsoP against B[*a*]P shows overall divergent associations between non-atopic and atopic OV/OB girls. Whereas the non-atopic OV/OB girls demonstrate an inverse association between 15-F_2t_-IsoP and B[*a*]P (Spearman’s rho = − 0.797; *p*-value = 0.010), the association is weakly null among the atopic OV/OB girls (Fig. 4).

**Fig. 5:**
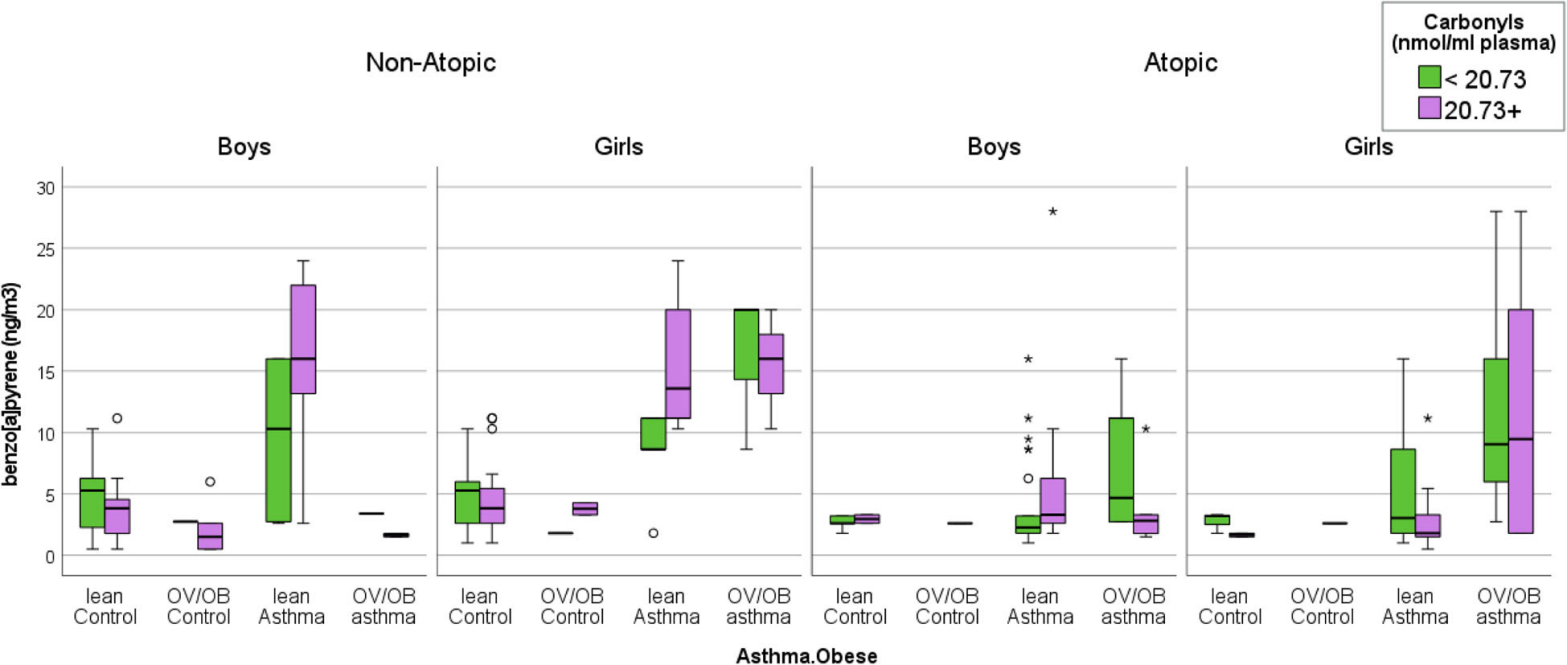
The box (i.e.*,* interquartile range and median) and whiskers (i.e., 5th and 95th percentile values) of ambient B[a]P concentrations for the health outcome groups (i.e., lean control, OV/OB control, lean asthma, and OV/OB asthma), in which each of the four outcomes are further stratified according to a median 8-oxo-dG value (5.68 nmol/mmol Creatinine). Each panel represents a combined group, according to the atopy status (atopic vs. non-atopic) and gender (boys vs. girls). The symbols O and * represent concentrations > 1.5– and > 3–fold of the 75th percentile value

When we alternatively stratify the same children, according to their overall median 8-oxodG (5.68 nmol/mmol Creatinine; Fig. 5), an overall similar trend as those in 15-F_2t_-IsoP is observed. Specifically, the non-atopic girls with a highest median B[*a*]P (20.0 ng/m^3^ IQR, 10.3-. ng/m^3^) and OV/OB asthma, or the second-highest median B[*a*]P (11.2 ng/m^3^ IQR, 8.6–18.0 ng/m^3^) and the lean asthma are observed among those children with lower than the median 8-oxodG, compared to the non-atopic controls (median, 3.3 ng/m^3^ IQR, 2.6–5.3 ng/m^3^). In contrast, the atopic girls with a highest median B[*a*]P (18.3 ng/m^3^ IQR, 6.7–28.0 ng/m^3^)-, as well as an OV/OB asthma, or the atopic girls with a second-highest median B[a]P (5.8 ng/m3 IQR, 1.6–12.4 ng/m3) and lean asthma, are observed among those with a higher than the median 8-oxodG, compared to the atopic control girls (median, 1.8 ng/m^3^ IQR, 1.8–1.8 ng/m^3^; Fig. 5).

Table 3 (B) summarizes the odds of multinomial asthma outcomes per unit increase in ambient B[a]P concentration in non-atopic and atopic boys and girls. An (ln)-unit increase B[*a*]P predicts approximately 10-times greater odds of asthma for the non-atopic boys with lean asthma (Table 3 (B)). Such estimate remains consistently robust, after controlling for Cotinine alone (aOR: 10.3; 95% CI: 3.2–33.1; *P < 0.001*; Table S1, Model A), Cotinine and lung function deficit (aOR: 9.0; 95% CI: 2.8 to 29.3; *P < 0.001*; Table S1, Model B), Cotinine, lung function deficit, and 15-F2t-IsoP (aOR: 9.4; 95% CI: 2.9 to 31.0; *P < 0.001*; Table S1, Model C), and finally, Cotinine, lung function deficit, 15-F2t-IsoP, and 8-oxodG (aOR: 9.6; 95% CI: 2.9 to 32.1; *P < 0.001*; Table S1, Model D), per unit exposure to B[*a*]P. In contrast, the same unit exposure to B[*a*]P among the non-atopic girls is associated with a dramatic reduction in asthma effect sizes following a stepwise adjustment for the same set of confounders (Table S1, Model A—D). For example, among the non-atopic girls with OV/OB asthma, the aOR changes from 301.2 (95% CI: 11.0–8231.9; *P = 0.001*) adjusting for cotinine (Table S1, Model A); to 139.7 (95% CI: 3.2–6120.7; *P = 0.010)* adjusting for cotinine and the lung function deficit (Table S1, Model B); to 71.2 (95% CI: 2.0–2563.5; *P = 0.020*) adjusting for cotinine, the lung function deficit, and 15-F_2t_-IsoP (Table S1, Model C); and to 46.1 (95% CI: 2.0–1271.4; *P = 0.024*) adjusting for cotinine, the lung function deficit, 15-F_2t_-IsoP, and 8-oxodG (Table 3 (B)).

On the other hand, the lean atopic children are not associated with elevated odds of asthma per unit change in B[*a*]P (Table 3). However, the only exception to such trend is observed among the atopic girls with OV/OB asthma, in whom a unit increase in B[*a*]P is associated with 5.9-times higher odds, following an adjustment for lung function deficit and Cotinine (95% CI: 1.1–31.3; *P = 0.038*); 7.6-times higher odds, following Cotinine, the lung function deficit, as well as 15-F_2t_-IsoP (95% CI: 1.2–49.1; *P = 0.034*); and 17.1-times higher odds, following an adjustment for Cotinine, the lung function deficit, 15-F_2t_-IsoP, and 8-oxodG (95% CI: 1.8–165.6; *P = 0.014*; Table 3 (B)).

### Sub-hypothesis 3: B[a]P association with the non-atopic OV/OB asthmatic girls are different from the B[a]P association with atopic OV/OB asthmatic girls

A unit increase in ambient B[*a*]P concentration is associated with 46.1-times greater odds of OV/OB asthma (95% CI: 1.7–1271.4; *P = 0.024*) among the non-atopic girls, adjusting for Cotinine, the lung function deficit, 15-F_2t_-IsoP, and 8-oxodG (Table 3 (B)). In contrast, the same unit increase in B[*a*]P among the atopic girls is associated with 17.1-times higher odds of OV/OB asthma following an adjustment for the same set of covariates (95% CI: 1.8–165.6; *P = 0.014*; Table 3 (B)).

## Discussion

Here, we characterize exposure to ambient B[*a*]P concentration and its role in non-atopic or atopic asthma as a proxy for non-Th2 and Th2-high endotypes, respectively. To the best of our knowledge, our analysis demonstrates for the first time that the childhood exposure level to B[a]P and the roles of two systemic oxidant markers, 15-F_t2_-isoP and 8-oxodG, are markedly divergent between the non-atopic asthmatic versus atopic asthmatic children. Our postulate of endotypes is further supported by the overall dissimilar pattern of co-morbid events during the children’s first three years of life, preceding the current asthma diagnosis. Namely, the so-called atopic march (e.g.*,* allergic rhinitis, upper respiratory infection) is absent among non-atopic asthmatic children. On the other hand, the atopic control children demonstrate the highest prevalence of the atopic march diagnoses.

Furthermore, contrary to the current body of evidence supporting adulthood onset of non-atopic asthma [37], our data suggest for the first time that the lung function deficit during early childhood a critical sentinel event preceding non-atopic asthma. Collectively, the following lines of evidence suggest that childhood exposures to B[*a*]P contribute toward non-atopic asthma. At the same time, the atopic one arises through B[*a*]P-independent mechanisms among the lean children.

First, among the lean children, B[*a*]P is not associated with elevated odds of atopic asthma, while it is associated with a robust increase in the odds of non-atopic asthma. For example, a unit B[*a*]P exposure is not associated with asthma among the lean atopic boys, while the same exposure predicts 10-times greater odds of asthma in the lean non-atopic boys. The non-atopic asthmatic boys with the highest exposure to B[*a*]P (median, 20 ng/m^3^) were also positively diagnosis with lung function deficit, compared to the non-atopic controls (median, 4.3 ng/m^3^). In contrast, in the lean atopic boys, median B[*a*]P was uniformly low in those with or without the deficit and with or without asthma. Such trends suggest that childhood exposure to elevated B[*a*]P level contributes toward the development of non-atopic asthma, while atopic asthma occurs through B[*a*]P-independent mechanisms in lean children.

Second, B[*a*]P is associated with plasma 15-F_2t_-IsoP and urinary 8-oxodG, respectively, in an opposite fashion between the children with non-atopic versus atopic asthma. While B[*a*]P and F_2t_-isoP are inversely associated among the non-atopic OV/OB girls, the same association is null among the atopic OV/OB girls (Fig. 4). Among the non-atopic lean girls, the same association is also weakly positive. Such non-linear dose-response suggests the ambient B[*a*]P concentration might pose a pro-inflammatory risk at low concentration, which subsequently switches to anti-inflammatory mechanism activation beyond a certain threshold B[*a*]P level [38]. Earlier investigations support hierarchical oxidative stress phases posed by multiple oxidants within air pollution exposures [39]. At low exposure concentration, the ambient B[*a*]P poses a pro-inflammatory risk, which subsequently switches to anti-inflammatory mechanism activation beyond a certain threshold [38]. Moreover, the adjustment for F_2t_-isoP and 8-oxodG, respectively, in the regression models is associated with significant decrements in B[a]P-asthma effect sizes non-atopic girls (Table 3). In contrast, the same covariate adjustments are associated with increased B[*a*]P-asthma associations among the atopic girls. Such divergent trends suggest that B[*a*]P might initiate and exacerbate distinct non-atopic versus atopic asthma mechanisms. Our earlier analyses have shown robust activation of anti-inflammatory mechanisms in children with high B[*a*]P exposure as well as a severe outcome [19]. Thus, while F_2t_-isoP and 8-oxodG concentrations seem suppressed among those who develop non-atopic asthma, the same oxidants appear to pose a mildly pro-inflammatory role in the atopic girls and corresponding increased odds of atopic OV/OB asthma (Table 3, Figs. 3 and 4).

Third, the question of whether OV/OB asthma represents a unique endotype or phenotypic consequence that remains unanswered [4, 10]. While multiple endotype might exist within so-called OV/OB asthma during adulthood, a unit B[*a*]P exposure is associated with vastly different estimates between non-atopic and the atopic girls within our case-control children (Table 3(B)). Among the non-atopic girls, a unit B[*a*]P exposure is associated with a step-wise increase in the odds of lean asthma (aOR, 27.4; 95% CI: 3.2 to 237.1) and OV/OB asthma (aOR, 46.1; 95% CI: 1.7 to 1271.4), respectively. In contrast, the same unit exposure is associated with markedly lower odds of OV/OB asthma (aOR, 17.1; 95% CI: 1.8 to 165.6) among the atopic girls. Overall, the B[*a*]P effect sizes differ more dramatically between the non-atopic and atopic children than between the lean and OV/OB children within either the non-atopic or atopic group. Our data suggest OV/OB asthma as a severe outcome, nested within the non-atopic asthma endotype, rather than constituting a unique endotype.

Fourth, the lung function deficit, which only appears among those with the highest median value of B[*a*]P, appears to be a particularly significant predictor of non-atopic asthma only. Overall, non-atopic boys with the highest median B[*a*]P exposure are associated with lung function deficit, as well as elevated odds of lean asthma. In contrast, the lean atopic boys and girls with low median B[*a*]P exposure are neither associated with the lung function deficit diagnosis nor asthma. Such a trend suggests that processes underlying non-atopic asthma might be distinct from those for atopic asthma among the boys. Lung function impairment appears to be a ‘meet-in-the-middle’ biomarker of B[*a*]P and asthma association. Above a certain threshold for exposure, B[*a*]P are associated with asthma, regardless of the atopy status. Even though spirometry-based lung function measurement is commonly considered a diagnostic criterion, Table 1 demonstrates that 81% of non-atopic asthmatic children and 76% of atopic asthmatic children are asthmatic without having lung function impairments. Several international diagnostic consensus statements noted that a considerable proportion of asthmatic children have normal lung function [40]. Instead, lung function impairment is significantly associated with severe, corticosteroid therapy-resistant asthma [3, 10, 41].

The strengths and limitations of the present study have been discussed [18–20, 23]. Briefly, B[*a*]P is used as a representative PAH compound here, while a more realistic exposure scenario involves exposure to a complex mixture of air pollutants. At the same time, a robust body of evidence, including our own, has demonstrated B[*a*]P as an etiologically pertinent and representative PAH compound [17, 18, 20, 23, 42–46]. Both the laboratory and epidemiologic evidence have shown that PAHs could induce or enhance allergic sensitization, exacerbate pre-existing asthma, and enhance the risk of de novo asthma development [44, 47, 48]. In particular, B[*a*]P has been shown to directly target hematopoietic stem cells by binding to aryl hydrocarbon receptors and subsequently impart a wide array of adverse effects, including mitochondrial functional deficit [49].

Furthermore, B[*a*]P represents an efficient indicator of a child’s exposure to ambient pollutant mixture due to its extremely high correlation with other traffic-related air pollutants [50–53]. At the same time, other constituents of complex mixtures could increase multiple types of oxidative injury, respiratory system inflammation, and alteration in lung structure and function [42]. Thus, other unmeasured yet correlated air pollutants (e.g.*,* metals) may pose a threat of residual confounding.

Our earlier investigation has shown the ambient monitored PAH concentrations as an apt marker of chronic exposure. For example, the interquartile range of ambient B[*a*]P levels during our exposure window of interest (i.e.*,* November 2008) is representative of the ambient concentrations from the earlier years [23]. However, using ambient monitoring as the primary marker of chronic exposure might underestimate exposure from other routes (e.g.*,* oral and dermal). Our earlier sensitivity analysis has shown that while both the dietary intake and the inhalation exposure to PAHs contribute to the human body burden, inhalation represents the predominant route of exposure during the heating season [54].

Another limitation of our investigation includes our lack of measurement for a full suite of cells and cytokines associated with Th 2 vs. non-Th2 endotypes. Thus, a more comprehensive characterization of the two asthmas in terms of the repertoire of cytokines, cells, and clinical traits of the children is warranted in future investigations. Also, as our goal was to estimate a proof-of-principle dose-response function of asthma associations under extreme variations in ambient B[*a*]P concentrations, our case-control children are not representative of the general Czech population.

## Conclusion

Within our case-control study, the non-atopic and atopic asthma are associated with distinct pathophysiologic processes, in which the non-atopic asthma is B[*a*]P-dependent, while the atopic one is B[*a*]P-independent. Elevated exposure to B[*a*]P is associated with depressed systemic oxidant levels and correspondingly elevated odds of non-atopic asthma. On the other hand, low ambient exposure to B[*a*]P, and the weakly pro-inflammatory effect of oxidative stress of such exposure, is not associated with atopic asthma

## Supplementary Information

**Additional file 1.**

## Data Availability

The data that support the findings of this study are available from the Institute of Experimental Medicine, Academy of Sciences of the Czech Republic, but restrictions apply to the availability of these data, which were used under license for the current study, and so are not publicly available. Data are, however, available from the authors upon reasonable request and with permission of the Institute of Experimental Medicine, Academy of Sciences of the Czech Republic.

## Abbreviations

PAH: Polyaromatic hydrocarbon
OV/OB: Overweight/obese
B[*a*]P: Benzo[*a*]pyrene
15-F_2t_-IsoP: 15-F_t2_-isoprostane
8-oxodG: 8-oxo-7,8-dihydro-2′-deoxyguanosine
JT: Jonkheere-Terpestra
IQR: Interquartile range
