# Supplementary material for: Airborne Benzo[a]Pyrene may contribute to divergent Pheno-Endotypes in children
Source: Environ Health. 2021 Apr 9;20:40. doi: 10.1186/s12940-021-00711-4 (PMC8035778; doi:10.1186/s12940-021-00711-4)
Supplement: Supplementary file 1 — Additional file 1. [file 12940_2021_711_MOESM1_ESM.docx]

**Online Supporting Material**

**Table S1:** Multinomial logistic regression models of ambient B[*a*]P, adjusting for cotinine (Model A), cotinine and lung function deficit (Model B), cotinine, lung function deficit, and 15-F_2t_-IsoP (Model C), and cotinine, lung function deficit, 15-F_2t_-IsoP, and 8-oxodG (Model D). Both active and second-hand smoke exposed children are excluded.

|  |  | Non-Atopic Children | | | |  | Atopic Children | | | |
| --- | --- | --- | --- | --- | --- | --- | --- | --- | --- | --- |
|  |  | Lean  Control | OV/OB  Control | lean  Asthma | OV/OB  asthma |  | Lean  Control | OV/OB  Control | lean  Asthma | OV/OB  asthma |
|  |  | N  aOR | N  aOR  (95% CI)  *P* | N  aOR  (95% CI)  *P* | N  aOR  (95% CI)  *P* |  | N  aOR | N  aOR  (95% CI)  *P* | N  aOR  (95% CI)  *P* | N  aOR  (95% CI)  *P* |
|  |  |  |  |  |  |  |  |  |  |  |
| Model A | Boys | 56  Ref. | 7  0.4  (0.1 to 1.0)  *P = 0.053* | **17**  **10.3**  **(3.2 to 33.1)**  ***P < 0.001*** | 3  0.5  (0.1 to 2.1)  *P = 0.335* |  | 7  Ref. | 1.0  0.7  (0.0 to 119.0)  *P = 0.910* | 43  2.0  (0.4 to 9.1)  *P = 0.361* | 12  2.6  (0.5 to 13.6)  *P = 0.245* |
|  | Girls | 55  Ref. | 3.0  0.3  (0.0 to 2.9)  *P = 0.311* | **11**  **43.0**  **(4.6 to 398.6)**  ***P = 0.001*** | **6**  **301.2**  **(11.0 to 8231.9)**  ***P = 0.001*** |  | 5  Ref. | 1.0  1.3  (0.0 to 121.4)  *P = 0.916* | 23  1.6  (0.5 to 5.7)  *P = 0.431* | **11**  **4.6**  **(1.0 to 21.2)**  ***P = 0.049*** |
|  |  |  |  |  |  |  |  |  |  |  |
| Model B | Boy | 56  Ref. | 7  0.4  (0.1 to 1.0)  *P = 0.053* | **17**  **9**  **(2.8 to 29.3)**  ***P < 0.001*** | 3  0.5  (0.1 to 2.1)  *P = 0.333* |  | 7  Ref. | 1  0.8  (0.0 to 270.3)  *P = 0.927* | 43  2.6  (0.4 to 16.1)  *P = 0.315* | 12  3.3  (0.5 to 23.5)  *P = 0.225* |
|  | Girl | 55  Ref. | 3  0.3  (0.0 to 2.9)  *P = 0.308* | **11**  **31.5**  **(3.1 to 316.7)**  ***P = 0.003*** | **6**  **139.7**  **(3.2 to 6120.7)**  ***P = 0.010*** |  | 5  Ref. | 1  1.6  (0.0 to 206.1)  *P = 0.844* | 23  2.2  (0.5 to 8.9)  *P = 0.279* | **11**  **5.9**  **(1.1 to 31.3)**  ***P = 0.038*** |
|  |  |  |  |  |  |  |  |  |  |  |
| Model C | Boy | 56  Ref. | 7  0.4  (0.1 to 1.0)  *P = 0.054* | **17**  **9.4**  **(2.9 to 31.0)**  ***P < 0.001*** | 3  0.3  (0.1 to 2.1)  *P = 0.238* |  | 7  Ref. | 1  0.8  (0.0 to 233.7)  *P = 0.936* | 43  2.6  (0.4 to 17.0)  *P = 0.330* | 12  3.5  (0.5 to 26.1)  *P = 0.222* |
|  | Girl | 55  Ref. | 3  0.2  (0.0 to 2.9)  *P = 0.262* | **11**  **30.6**  **(3.1 to 303.0)**  ***P = 0.003*** | **6**  **71.2**  **(2.0 to 2563.5)**  ***P = 0.020*** |  | 5  Ref. | 1  1.4  (0.0 to 1049.1)  *P = 0.928* | 23  2.4  (0.5 to 11.5)  *P = 0.288* | **11**  **7.6**  **(1.2 to 49.1)**  ***P = 0.034*** |
|  |  |  |  |  |  |  |  |  |  |  |
| Model D | Boy | 56  Ref. | 7  0.3  (0.1 to 1.0)  *P = 0.054* | **17**  **9.6**  **(2.9 to 32.1)**  ***P < 0.001*** | 3  0.3  (0.1 to 2.1)  *P = 0.247* |  | 7  Ref. | 1  0.4  (0.0 to 173.1)  *P = 0.786* | 43  1.6  (0.3 to 9.7)  *P = 0.594* | 12  2.2  (0.3 to 14.9)  *P = 0.409* |
|  | Girl | 55  Ref. | 3  0.3  (0.0 to 2.9)  *P = 0.286* | **11**  **27.4**  **(3.2 to 237.1)**  ***P = 0.003*** | **6**  **46.1**  **(1.7 to 1271.4)**  ***P = 0.024*** |  | 5  Ref. | 1  0  (0.0 to 0.0)  *P = 0.998* | 23  3.6  (0.6 to 23.3)  *P = 0.173* | **11**  **17**  **(1.8 to 165.6)**  ***P = 0.014*** |
